# *Cronobacter* Infections Not from Infant Formula, Taiwan

**DOI:** 10.3201/eid1901.120774

**Published:** 2013-01

**Authors:** Hsih-Yeh Tsai, Chun-Hsing Liao, Yu-Tsung Huang, Ping-Ing Lee, Po-Ren Hsueh

**Affiliations:** Author affiliations: Far Eastern Memorial Hospital, New Taipei City, Taiwan (H.-Y. Tsai, C.-H. Liao, Y.-T. Huang);; National Taiwan University College of Medicine, Taipei, Taiwan (H.-Y. Tsai, Y.-T. Huang, P.-I. Lee, P.-R. Hsueh)

**Keywords:** Cronobacter sakazakii, infection, Taiwan, bacteria, adults, immunocompromised, infant formula

**To the Editor:** Species of the genus *Cronobacter* are relatively heterogeneous at the phenotypic and molecular levels (*1*). In 2012, the following 7 *Cronobacter* species had been defined: *C. sakazakii, C. malonaticus, C. turicensis, C. dublinensis, C. muytjensii*, *C. condimenti,* and *C. universalis* (*2*). These opportunistic pathogens cause bacteremia and meningitis in neonates and are associated with necrotizing enterocolitis (*3*); ≈30% of infants with *Cronobacter* bacteremia or meningitis have died (*4*). *Cronobacter* spp. primarily infect infants, but infections among immunocompromised patients, particularly elderly patients, have been reported (*5*). Although these organisms are ubiquitous in the environment and have been isolated from a variety of foods, *Cronobacter* spp. infections in infants have been epidemiologically associated with ingestion of contaminated powdered infant formula (*6*). Few reports of *C. sakazakii* infections in adults have been published.

During 2002–2011, a total of 5 *C. sakazakii* isolates, 1 from each of 5 patients, were identified at the National Taiwan University Hospital in northern Taiwan (Table). These isolates were identified as belonging to the *C. sakazakii* group by use of 2 systems: PMIC/ID-30 (Becton Dickinson Diagnostic Systems, Sparks, MD, USA) and the Vitek 2 System GN card (bioMérieux Inc., La Balme les Grottes, France). The phenotypic profiles that use 14 biochemical characteristics to differentiate 7 species and 3 subspecies of *C. dublinensis* within *Cronobacter* gen. nov. (*C. sakazakii* group) have been described (*2*). Although we did not apply the 14 biochemical tests to differentiate the 5 isolates (*2*), the isolates’ lack of indole production and dulcitol utilization, obtained by use of Enterotube II (Becton Dickinson Diagnostic Systems), was compatible with identification of the following 3 species or subspecies: *C. sakazakii, C. malonaticus,* or *C. dublinensis* subsp. *lausannensis* (*2*)*.* Results of partial 16S rRNA gene sequence analysis with primers 8FPL and 1492RPL indicated that the isolates were probably *C. sakazakii* (*7*), and results of a 2-step *rpoB*-based PCR that used 2 sets of primer pairs (*Csakf/Csakr* and *Cmalf/Cmalr*) confirmed that the isolates were *C. sakazakii* (*8*)*.*

**Table:** Characteristics of 5 patients with *Cronobacter sakazakii* infections, National Taiwan University Hospital, Taiwan, 2002–2011*

Patient age/sex	Year of diagnosis	Underlying medical conditions	Clinical presentation	Infection type	Outcome	Isolation site	Isolate GenBank accession no.	Isolate serogroup
77 y/M	2002	Laryngeal cancer, diabetes mellitus, pulmonary tuberculosis	Cardiac arrest at arrival at emergency department	Primary bacteremia	Died	Blood	FJ947061.1	O1
72 y/M	2002	Gastric cancer	Fever	Primary bacteremia	Survived	Blood	JF330153.1	NT
2 mo/M	2005	Congenital heart disease	Fever	Pneumonia	Survived	Sputum	F330133.1	O1
37 y/M	2008	None	Abdominal pain	Acute cholecystitis	Survived	Bile	JF330153.1	NT
64 y/F	2011	Breast cancer	Hemoptysis	Pneumonia	Survived	Sputum	GU727684.1	NT

*All isolates were identified as C. *sakazakii* I (99% identity) by 16S sequencing. NT, not typeable (not serogroups O1, O2, or O3).

Serogroups of the 5 *C. sakazakii* isolates were determined by using 5 primer pairs specific to the *weh*C, *weh*I, and *wzx* genes (*9*). Of these 5 isolates, 3 were serogroup O1, and 2 were not typeable (not serogroups O1, O2, or O3).

Results of disk-diffusion susceptibility testing showed that all isolates were susceptible to cefotaxime, cefepime, piperacillin–tazobactam, ertapenem, imipenem, meropenem, ciprofloxacin, gentamicin, and amikacin and that all were resistant to cefazolin. The random amplified polymorphic DNA patterns generated by arbitrarily primed PCR that used 2 random oligonucleotide primers (M13 and ERIC1) differed among the 5 isolates, indicating that these 5 *C. sakazakii* strains were not clonally related (*10*).

The clinical and microbiological characteristics of the 5 patients (4 adult, 4 male) with *C. sakazakii* infection are summarized in the Table. Primary bacteremia was found in 2 patients, pneumonia in 2 (predominant growth of *C. sakazakii* from purulent sputum samples), and acute cholecystitis in 1.

The nonadult patient was a 2-month-old boy with congenital heart disease. Because of apnea and cyanosis, he was sent to an emergency department and later received assisted ventilation and supportive care in an intensive care unit. He was extubated on day 11 of hospitalization; however, fever and increased purulent sputum were noted on day 18. Bacterial culture of the suctioned sputum specimen yielded *C. sakazakii.* Before being hospitalized, the boy had been fed reconstituted powdered infant formula (Nestlé H.A.1, Gold; Nestlé Taiwan Ltd., Taipei, Taiwan) by mouth without other supplemental nutrition. During hospitalization, he received infant formula made by the hospital nutritional department through nasogastric tube. Although the powdered infant formula was not tested for *C. sakazakii,* initial sputum culture disclosed viridans group streptococci, and *C. sakazakii* was isolated from sputum obtained on day 18 of hospitalization. Thus, *C. sakazakii* from this infant might not have been associated with contaminated powdered infant formula.

Among the 4 adult patients, 3 had underlying solid organ malignancy and had received immunosuppressive drugs., and the other had bacteremia and died of cardiac arrest at arrival at the emergency department. The sources of *C. sakazakii* infection in the 1 nonimmunocompromised adult and the infant remain unclear; further research is needed to identify the source of *C. sakazakii* infections in Taiwan.
